# The interaction between nanoparticles and immune system: application in the treatment of inflammatory diseases

**DOI:** 10.1186/s12951-022-01343-7

**Published:** 2022-03-12

**Authors:** Jin Liu, Zeyang Liu, Yan Pang, Huifang Zhou

**Affiliations:** 1 Department of Ophthalmology, Shanghai Ninth People’s Hospital, Shanghai Jiaotong University School of Medicine, Shanghai, 200011 China; 2 Shanghai Key Laboratory of Orbital Diseases and Ocular Oncology, Shanghai, 200011 China

**Keywords:** Nanoparticles, Immune system, Immune cells, Immune molecules, Inflammatory diseases

## Abstract

Nanoparticle (NP) is an emerging tool applied in the biomedical field. With combination of different materials and adjustment of their physical and chemical properties, nanoparticles can have diverse effects on the organism and may change the treating paradigm of multiple diseases in the future. More and more results show that nanoparticles can function as immunomodulators and some formulas have been approved for the treatment of inflammation-related diseases. However, our current understanding of the mechanisms that nanoparticles can influence immune responses is still limited, and systemic clinical trials are necessary for the evaluation of their security and long-term effects. This review provides an overview of the recent advances in nanoparticles that can interact with different cellular and molecular components of the immune system and their application in the management of inflammatory diseases, which are caused by abnormal immune reactions. This article focuses on the mechanisms of interaction between nanoparticles and the immune system and tries to provide a reference for the future design of nanotechnology for the treatment of inflammatory diseases.

**Graphical Abstract:**

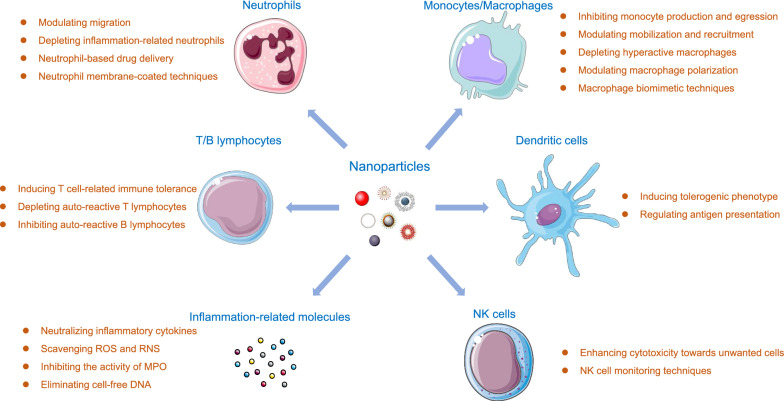

## Background

The past decade has witnessed the rapid development of nanotechnology, especially its application in the medical field. Since Doxil^®^, a PEGylated liposomal doxorubicin formulation became the first US Food and Drug Administration (FDA) approved nano-drug in 1995, nanotechnology has drawn great interest in the development of novel theranostic strategy [1]. In recent years, people have recognized that nanoparticles (NPs) within 1–100 nm have their unique application in biomedical field due to the ingenious combination of their salient physical and chemical characters and proper interaction with the constituent elements of life at nanoscale [2]. Generally, nanoparticles can be classified into two types based on their chemical composition: organic and inorganic nanoparticles. Organic nanoparticles mainly include polymers, exosomes, liposomes, protein-based nanoparticles etc., while inorganic nanoparticles consist of silica nanoparticles, metal nanoparticles, carbon nanotubes, quantum dots and so forth [2, 3]. But in some cases, a more complex structure and combination of different chemical materials will be needed to achieve combined functions. For example, metal cores will be applied for physical properties, together with (bio)organic coating on the surface to maintain stabilization of the structure in host body [4]. Apart from the chemical constitutions bringing their inherent properties, physical characteristics of NPs like size, shape, elasticity, surface charge and surface functionalization can also significantly shape their functions in the body, endowing unlimited potential for the application of nanotechnology in biomedicine [5]. Nowadays, NPs have been implicated for its application in multiple cancers, autoimmune diseases, bone regeneration etc. and dozens of formulas are under experimental and clinical trials [2, 6, 7].

The immune system is responsible for the recognition and subsequent neutralization or elimination of foreign entities [8]. It’s now well-accepted that abnormal immune system is involved in all kinds of inflammatory diseases, degenerative disease as well as benign and malignant tumors. The immune response can be divided into two parts, namely innate immunity and adaptive immunity in terms of speed and specificity of the reaction [9]. Innate immunity is acquired at birth, it encompasses the pre-deployed cellular and molecular components that can immediately protect the host organism against the pathogen and therefore serve as the first line of defense of the immune system [10]. Once the physical barriers of the body like skin, mucosa etc. are breached, the pre-existing effectors and soluble mediators come into action, such as lysozyme, C reactive protein, mannan-binding lectin etc. Then a variety of cell types participate in the innate immune responses as a second line of defense against pathogens, including phagocytes [e.g., macrophages, neutrophils, dendritic cells (DCs)], and leukocytes [e.g., natural killer (NK) cells, mast cells, eosinophils, basophils] [8]. If the pathogens are not completely eliminated by innate immunity and enter the peripheral lymphatic organs and tissues, the protective role of adaptive immune response comes into play as the third line of defense [11]. Different from innate immunity, the adaptive immunity is more accurate with specific antigen stimulation being an essential condition, but is relatively slow which usually happens 96 h after the invasion of pathogens. Besides, adaptive immunity can be important during secondary infections due to its capacity to “remember” and respond more effectively to restimulation [12]. Such immune response is mainly induced by T and B lymphocytes, and can be divided into two types: cell-mediated and humoral adaptive immunity. Cell-mediated immunity mainly involves cytotoxic T cells that can recognize affected cells and induce cell death, while the latter type of immunity is established by CD4^+^ helper T cells via activation of B cells which produce specific antibodies against the pathogen [13, 14].

Inflammatory diseases are a group of conditions caused by abnormal immune reaction, which results in local or systemic inflammation. Currently, treatment for inflammatory diseases mainly includes anti-inflammatory drugs which could help attain the balance of inflammatory and immune responses [15]. However, traditional anti-inflammatory agents are faced with obstacles like low tissue specificity, systemic side effects and inefficiency in crossing biological barriers, which proposes the need for novel and more efficient anti-inflammatory strategies. Recent studies focus on hybrid materials that have anti-inflammatory effects together with better specificity. This review aims to summarize recent advances in the development of NPs that could interact with the immune system, including different cellular components and inflammation-related molecules. We’ll discuss the application of NPs in the treatment of different kinds of inflammatory diseases, hoping to provide suggestions for future nanotool research and development.

## The interaction between NPs and the immune system

**Fig. 1:**
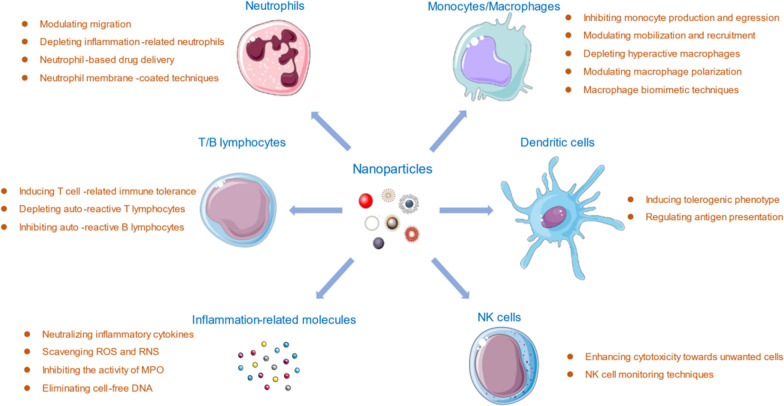
Schematic illustration of interaction between NPs and different components of the immune system in managing inflammatory diseases

The immune system involves immune organs, immune cells and immune molecules. Presently, NP formulas mainly influence the immune system through specific interactions with a variety of immune cells and molecules. NPs can be designed targeting the cellular and molecular components based on their own characters and exert accurate modulating functions, which forms the theoretical basis for NP-based therapy. In this section, we will discuss the design of NP strategies and their interplay with different components of the innate and adaptive immune system, especially focused on the application in the treatment of inflammatory diseases (Fig. 1).

### The interaction between NPs and monocytes/macrophages

Macrophages are important cellular components of innate immune system. They can be found in all kinds of tissues and are extremely heterogeneous and plastic with unique roles in almost every aspect of an organism’s biology ranging from development, homeostasis, tissue repair, and immunity [16, 17]. The origin of macrophages is rather complex. Previously, it can be explained by the widely-accepted mononuclear phagocytic system (MPS) theory, in which mature macrophages are defined as the end cells of the mononuclear phagocytic lineage, with tissue macrophages deriving from circulating monocytes that originate form CD34^+^ hematopoietic stem cells in the bone marrow [16]. However, this long held-view has been challenged in recent years by evidence that in many organs like brain, liver, kidney, lung and heart, macrophages originate from the yolk sac or fetal liver and their maintenance in adulthood in a homeostatic status is independent of circulating monocytic precursors, while in tissues like the gut, the dermis, and the heart, circulating monocytes contribute to macrophage populations [18]. Based on the evidence available today, it can be speculated that human body possesses a dual origin of tissue macrophages (self-sustaining local versus monocyte-derived), as illustrated by heart macrophages [19]. Besides, macrophages can be further classified according to their different ways of activation and function characteristics as classically activated (or inflammatory) macrophages (M1) and alternatively activated (or wound‐healing) macrophages (M2) [20]. M1 cells are pro-inflammatory and polarized by lipopolysaccharide (LPS) either alone or in association with T-helper 1 (Th1) cytokines such as Interferon-γ (IFN-γ), granulocyte–macrophage colony-stimulating factor (GM-CSF). They have a stronger ability of antigen presentation and can produce pro-inflammatory cytokines such as interleukin (IL)-1β, IL-6, IL-12, IL-23, and Tumor necrosis factor α (TNF-α) [21]. On the contrary, M2 cells are anti-inflammatory and immunoregulatory. They are activated by T-helper 2 (Th2) cytokines such as IL-4 and IL-13 and produce anti-inflammatory cytokines such as IL-10 and transforming growth factor-β (TGF-β) [20–22]. The polarization of M1/M2 macrophages actually modulates the homeostasis of pro-inflammatory and anti-inflammatory reaction in our body, and though not fully understood, such “activation” mechanism has been recognized to play a key role in a variety of physiology process as well as pathology like inflammatory disease, pathological pregnancy, infection and cancer [17, 21].

Currently, given the key roles monocytes/macrophages play in multiple pathogenic process, a variety of nanoparticle formulas have been designed to interact with these cellular components [23]. To specifically target macrophages and achieve these functions at the same time, nanoparticles need to be decorated with different ligands on the surface, including monoclonal antibodies, peptides, oligomers or small molecules like mannose, legumain etc., [24], they can bind to certain receptors that are over-expressed on the surface of macrophages. The effects of nanoparticles on monocytes/macrophages range from modulating bone marrow activation, monocytes mobilization and recruitment to microvascular permeability enhancement, polarization modulation etc. Nanoparticles can also utilize the natural characters of monocytes/macrophages to enhance their targeting effectiveness and avoid immune system clearance at the same time (Table 1).

**Table 1:** Interaction between nanoparticles and monocytes/macrophages

Carrier	Cargo	Function	Diseases	References
Liposome	siRNA	Inhibiting monocyte production and egression in the bone marrow	Atherosclerosis, myocardial infarction, pancreatic islet transplantation in diabetes	[29]
*L*-glutamic acid, *N*-(3-carboxy-1-oxopropyl)-, 1,5-dihexadecyl ester modified liposome	–	Bone marrow accumulation	–	[31]
EGFP-EGF1-conjugated PLGA NPs	shRNA	Modulating macrophage recruitment	Atherosclerosis	[38]
Lipid–polymer NPs	siRNA	Modulating monocyte recruitment	Myocardial infarction	[39]
PLGA NPs	Irbesartan	Modulating monocyte recruitment	Myocardial ischemia–reperfusion injury	[40]
7C1 NPs	siRNA	Modulating monocyte recruitment	Myocardial infarction	[42]
α-gal nanoparticles	–	Modulating macrophage recruitment	Myocardial infarction	[44]
α-gal nanoparticles	–	Modulating macrophage recruitment	Diabetic wound	[45]
Liposomes	Clodronate	Macrophage depletion	Endometriosis	[52]
Liposomes	Clodronate	Macrophage depletion	Rheumatoid arthritis	[53]
Liposomes	Clodronate	Macrophage depletion	Rheumatoid arthritis	[54]
Dendrimer	Methotrexate	Macrophage depletion, anti-inflammation	Rheumatoid arthritis	[56]
Reconstituted high-density lipoprotein NPs	Statins	Inhibiting macrophage proliferation	Atherosclerosis	[57, 58]
Mesoporous silica NPs	IL-4	Modulating macrophage polarization	–	[62]
Gold NPs	–	Modulating macrophage polarization	Bacterial sepsis	[63]
Hyaluronic acid-poly(ethyleneimine) (HA-PEI) NPs	IL-4/IL-10 expressing plasmid DNA	Modulating macrophage polarization	LPS-induced inflammation	[64]
HA-PEI NPs	MicroRNA-223	Modulating macrophage polarization	LPS-induced inflammation	[65]
Graphene oxide NPs	IL-4 expressing plasmid DNA	Modulating macrophage polarization	Myocardial infarction	[66]
Macrophage plasma membrane coated liposomes	Dexamethasone	Selective targeting inflamed tissues	Phlogosis	[69]
Macrophage cell membrane coated PLGA NPs	–	Neutralize endotoxins and proinflammatory cytokines	Sepsis	[79]
Polyion complex NPs	Catalase	Anti-inflammation and improving neuroprotective ability	Parkinson's disease	[73]
Iron oxide NPs	–	Monocyte imaging with MRI	Cutaneous inflammation	[75]
Poly(lactide-coglycolide) NPs	–	Modulating macrophage polarization	Spinal cord injury	[80]

#### Inhibiting monocyte production and egression in the bone marrow

Bone marrow activation is one of the major characters of inflammatory disorders. Under inflammatory conditions, elevated levels of inflammatory cytokines, Toll-like receptors (TLR) agonists and noradrenaline will cause the proliferation of hematopoietic stem cells (HSCs), leading to overproduction of inflammatory monocytes, which will accumulate in inflammatory lesions [25, 26]. Besides, in the bone marrow environment, chemokine C–C motif ligand 2 (CCL2)/chemokine C–C motif receptor 2 (CCR2) interaction and the increase in permeability of blood vessels will enhance the motivation/egression of inflammatory monocytes [27]. Therefore, NPs can be designed to target different stages of inflammatory monocytes production and egression for the diagnosis and treatment of inflammatory diseases. For example, corticosteroids [28] and CCR2 small interfering RNA (siRNA) [29] loaded NPs have been designed respectively to modulate the response of bone marrow under inflammatory conditions. Such interference significantly reduced the number of monocytes accumulated in sites of inflammation and suppressed the progress of inflammatory diseases like atherosclerosis, myocardial infarction and pancreatic islet transplantation in diabetes [29]. Efforts have also been made to develop strategies of enhancing nanoparticles accumulation in the bone marrow. Bisphosphonates (like alendronate) conjugated to different types of nanoparticles can increase their targeting affinity to hydroxyapatite, major mineral component of the bone [30]. Synthetic substances, like the anionic amphiphilic; *l*-glutamic acid *N*-(3-carboxy-1-oxopropyl)-1,5-dihexadecyl ester, can increase the accumulation of liposomes in the bone marrow and inhibit hepatic uptake concomitantly [31]. In addition, therapeutic effects of nanoparticles on monocyte production in the bone marrow can be assessed through molecular imaging approaches. For instance, the proliferation of bone marrow monocytes and HSCs can be quantified through ^18^F-3′-fluoro-3′-deoxy-*l*-thymidine-PET (^18^F-FLT-PET) [32, 33].

#### Modulating monocyte/macrophage mobilization and recruitment

NPs can also be designed to modulate the process of monocyte/macrophage mobilization and recruitment. During the course of chronic inflammation, monocytes/macrophages can be mobilized and recruited through a variety of chemokines like CCL2 (also called monocyte chemotactic protein 1, MCP-1) and chemokine C–C motif ligand 7 (CCL7) [34, 35], and receptors like angiotensin II (Ang II) receptors [35], then accumulate in the region of inflammation [36, 37], so NPs targeting such molecules can inhibit the mobilization and recruitment process of inflammatory monocytes/macrophages and play an anti-inflammatory role. Wu et al. developed a CCR2 short hairpin (shRNA)-loaded EGFP-EGF1-conjugated poly (lactic-co-glycolic acid) (PLGA) nanoparticles (ENPs) to selectively knock down the expression of CCR2 in atherosclerotic cellular models of macrophages, which becomes a potential tool for modulating macrophage-related inflammation in atherosclerosis [38]. A similar strategy with MCP-1 siRNA-loaded lipid–polymer NPs successfully inhibited the mobilization and recruitment of inflammatory monocytes to the diseased heart from haematopoietic niche in a mouse model of myocardial infarction [39]. Moreover, bioabsorbable PLGA nanoparticles incorporating irbesartan have been designed to inhibit the recruitment of inflammatory monocytes to the heart through blocking the angiotensin II type 1 receptor in a mouse model of myocardial ischemia–reperfusion (IR) injury [40]. Monocyte-endothelial adhesion, a major part of monocyte recruitment process, is considered critical initiator of inflammatory vascular diseases like atherosclerosis [41], and a variety of adhesion molecules have been identified to play important roles in such process. Based on this, delivering siRNA-loaded nanoparticles to knock down 5 different cell adhesion molecules has shown to significantly reduce monocyte recruitment into atherosclerotic lesions and decreased matrix-degrading plaque protease activity after myocardial infarction in mice, thereby reducing local inflammation of infarcted myocardium and improving recovery after ischemia [42].

The impaired healing of tissue damage caused by inflammatory diseases like diabetes and myocardial infarction is another serious clinical problem bringing huge public health burden, the mechanism of which has been linked to an inadequate response from macrophages [43–45]. The α-gal epitope (with the structure as Galα1-3Galβ1-4GlcNAc-R) is a carbohydrate antigen synthesized in non-primate mammals, lemurs, and New-World monkeys [46]. It can bind to its natural antibody, which are abundant in human body, and forms immune complexes that can recruit antigen-presenting cells (APCs) such as macrophages [46]. Galili et al. utilized α-gal nanoparticles prepared from rabbit red blood cell membranes to recruit pro-reparative macrophages to the infarcted territory in a mouse model of myocardial infarction. After 28 days of treatment, treated mice demonstrated a marked remission in terms of infarct size (~ tenfold smaller), restoration of normal myocardium structure and contractile function comparing with control group [44]. Such strategy has also been applied to manage diabetic wound healing with satisfying outcomes [45].

#### Depleting hyperactive macrophages

Lesional inflammation can not only be triggered by circulating monocytes recruitment, but can also be driven by local macrophage proliferation [47–49], thus inhibiting the recruitment process alone may not be enough to control local inflammation, especially in the case that the inflammatory condition has been established [49, 50]. Multiple nanoparticle products have been developed to restricting the proliferation of focal macrophages or directly depleting them to reduce the burden of local inflammation. Clodronate liposomes have become the agent of choice for macrophage depletion in a variety of diseases models for many years [51]. Bacci et al. utilized clodronate liposomes for the depletion of macrophages in mouse models of endometriosis [52]. Clodronate liposomes were i.p. injected at different time points after implantation of the endometrial tissue in recipient mice. In the treatment group, clodronate liposomes significantly reduced the number of F4/80^+^ and CD11b^+^ macrophages in the peritoneum of sacrificed animals, accompanied by a significant reduction in the weight of endometriotic lesions. Researchers have also utilized clodronate liposomes to deplete synovial macrophages in mice with rheumatoid arthritis (RA), which resulted in reduced inflammation and prevented joint destruction [53, 54]. Another exploration to alleviate RA inflammation is Methotrexate conjugated dendrimers. Methotrexate is a folate inhibitor with anti-proliferative and anti-inflammatory activities [55]. Such nano-formula could specifically target the inflammation-activated, folate receptors overexpressing macrophages and successfully reduced ankle swelling, paw volume, cartilage damage, and bone resorption in rat models of RA [56]. Furthermore, reconstituted high-density lipoprotein (rHDL) nanoparticles encapsuled with statins have been applied in mouse models of atherosclerosis [57, 58]. Plaque macrophage content was significantly reduced and plaque inflammation progression was inhibited in the treating group.

#### Modulating macrophage polarization

**Fig. 2:**
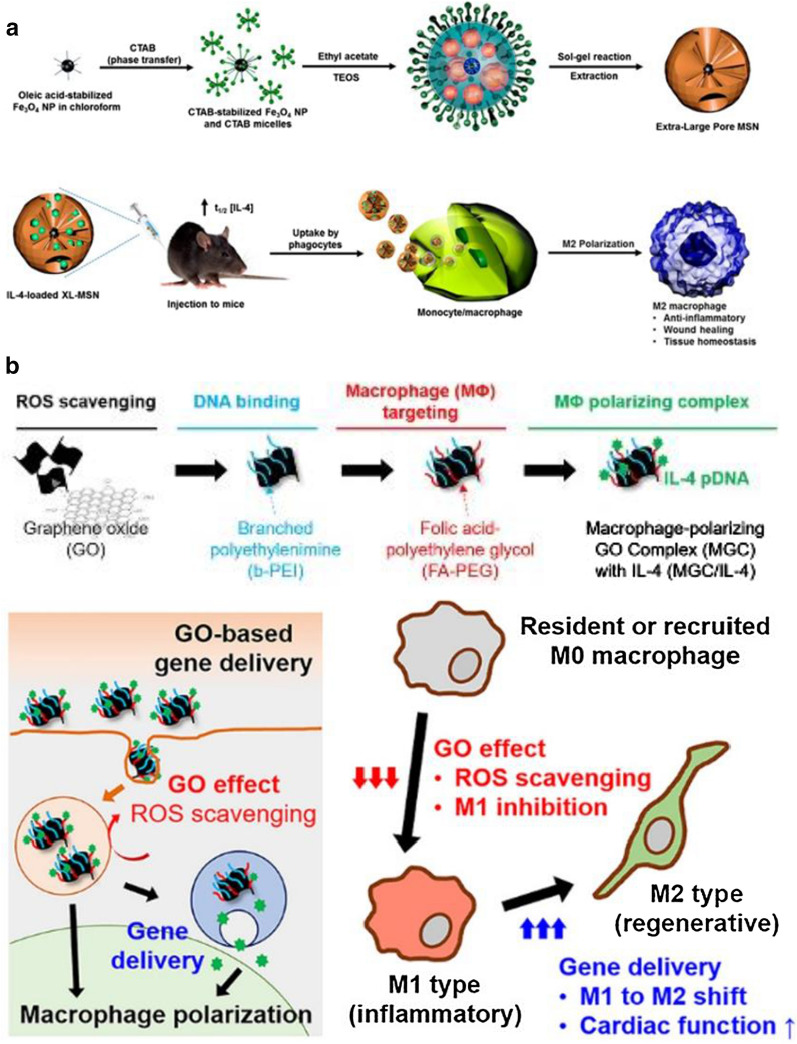
NP strategies that can modulate macrophage polarization in managing inflammatory diseases. **a** Schematic illustration of synthesis process of mesoporous silica nanoparticles with extra-large pores (XL-MSNs) and their effects of M2 macrophage polarization through IL-4 delivery. Reprinted with permission [62]. Copyright 2017 American Chemical Society. **b** Preparation process of macrophage-polarizing graphene oxide (GO) complex (MGC)/IL-4 pDNA and their modulating effects of macrophage polarization. Reprinted with permission [66]. Copyright 2018 American Chemical Society

As described before, macrophage polarization plays a pivotal role in the process of inflammation, especially in the pathogenesis of multiple inflammatory diseases. With different stimuli from the inflammatory microenvironment, macrophages can polarize into either inflammatory type (M1) or wound-healing type (M2). M1 type cells attract many other cell types at the inflamed site via pro-inflammatory cytokines and chemokines and ultimately worsen the situation in inflamed tissue [59], while M2 cells acts the opposite, with anti-inflammatory cytokines released and inflammatory status will be relieved. Based on such theory, a number of nanoparticle formulas have been developed to modulate macrophage polarization more towards M2 type, while restricting M1 macrophage population at the same time [60, 61]. Strategies include cytokines delivery, metal nanoparticles, plasmid DNA/microRNA delivery etc. Kwon et al. have designed mesoporous silica nanoparticles with extra-large pores (XL-MSNs) to deliver IL-4, an M2-polarizing cytokine, to macrophages [62]. Results showed that the injection of IL-4 loaded XL-MSNs triggered the macrophage M2 polarization in vivo, indicating a potential of targeted delivery of cytokines for modulating macrophage polarization (Fig. 2a). Metallic nanoparticles have also been applied in modulating the macrophage polarization process. Gold nanoparticles (AuNP) have several biochemical advantageous properties and show anti-inflammatory effects in mice. Researchers administrated AuNP as an adjuvant to antibiotics in a mouse cecal ligation and puncture model of bacterial sepsis. Results showed that AuNP could decreased M1 macrophages (CD86 + ve in F4/80 + ve cells) and increased M2 macrophages (CD206 + ve in F4/80 + ve cells) in the spleens of sepsis mice, such effects were further proved by in vitro experiments [63]. Apart from that, several synthetic immunomodulatory nanoparticles have been designed to deliver plasmid DNA or microRNA to re-polarized macrophages. Multiple hyaluronic acid-poly (ethyleneimine) NPs (HA-PEI) encapsulated IL-4/IL-10 expressing plasmid DNA, or microRNA-223, have been applied to modulate the reprogramming of macrophages from the M1 to M2 subtype, confirmed by increased Arg level, decreased iNOS level, higher expression of CD206, and down-regulation of the M1 marker CD86 etc., [64, 65]. Also, graphene oxide (GO) nanoparticles carrying IL-4 expressing plasmid can achieve the similar polarization modulating effects [66] (Fig. 2b). These results indicated the possibility of delivering nucleic acid for the modulation of macrophage polarization.

#### Macrophage biomimetic techniques

To achieve their therapeutic efficacy, nanoparticles need to escape from the immune system, cross the biological barriers of the body and finally reach the target tissues [67], which involves a wide range of complex functions. Instead of developing such functions through synthetic techniques, researchers are now directly leveraging naturally derived cell membranes as a means of bestowing nanoparticles with enhanced biointerfacing capabilities [68], with monocytes/macrophages as an important source of cell-derived membranes. Parodi et al. [67] designed nanoporous silicon nanoparticles coated with cellular membranes purified from leukocytes. Such hybrid nanoparticles are able to evade opsonization and reduce phagocytosis of the immune system, communicate with endothelial cells through receptor-ligand interaction, transport and release their payload across an inflamed reconstructed endothelium, which implicates a potential for their application in treating inflammatory diseases and tumor. Molinaro et al. [69] described a method that incorporate proteins derived from the macrophage plasma membrane into lipid nanoparticles. The resulting proteolipid vesicles preferentially targeted inflamed vasculature enabled the selective and effective delivery of dexamethasone to inflamed tissues, and reduced phlogosis in a localized model of inflammation. Apart from these, an alternative approach is cellular-hitchhiking or backpacking, which is achieved by designing nanoparticles attached to the surface of or embedded in monocytes/macrophages [70, 71]. Such formula utilizes the natural abilities of circulatory cells to target inflamed tissues while avoiding immune system clearance and has been exploited in the management of inflammation-related diseases like Parkinson's disease and virus infection [72, 73].

#### Monocyte/macrophage cell tracking

**Fig. 3:**
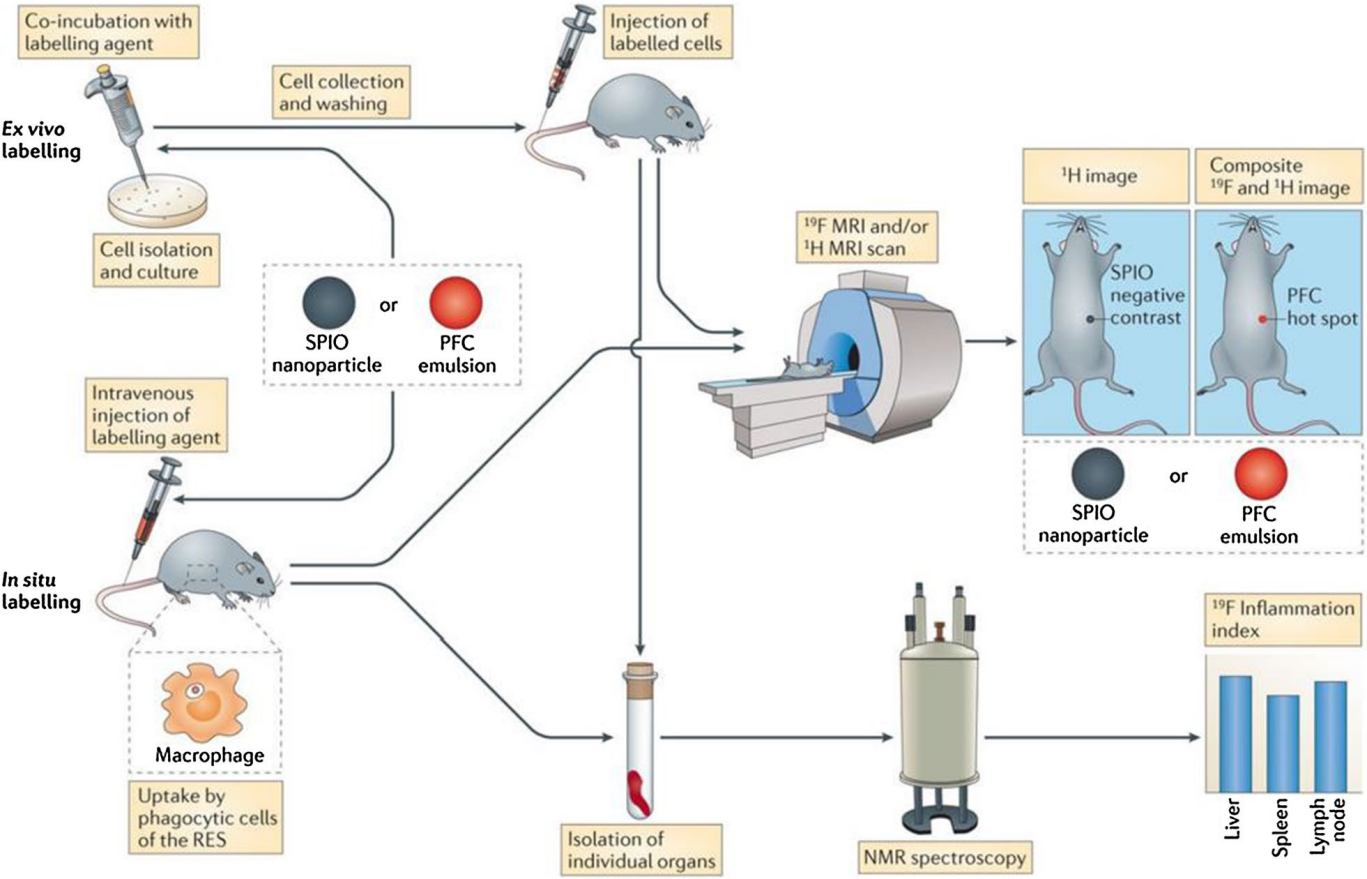
Cell tracking technique can be used to monitor monocyte dynamics. Cells are selected and incubated with labelling reagent in vitro—superparamagnetic iron oxide (SPIO) nanoparticles or perfluorocarbon (PFC) emulsion, then these cells are administered to the body. The labelling agent can also be directly intravenously injected and taken up by monocytes/macrophages, which then accumulated in the inflamed region. Both in vitro and in vivo labelling cells can be monitored through ^1^H magnetic resonance imaging (MRI) or ^19^F MRI scan. In addition, ^19^F MRI signal can be quantified by nuclear magnetic resonance (NMR) spectroscopy to evaluate inflammation severity. Reprinted with permission [74]. Copyright 2013 Springer Nature

Non-invasive cell tracking is an emerging approach for imaging cells in their native environment [74]. Such strategy could not only allow us to evaluate disease progression and response to treatment, but also help to design immunomodulatory therapeutics that utilize monocyte features to increase their tissue/lesion penetration [34]. Currently, several imaging techniques combined with nanotechnology have been applied to monitor monocyte movement. For example, magnetic resonance imaging (MRI) has been widely used to study monocyte dynamics in deeper tissues given its excellent spatial resolution. Contract MRI uses different probes like superparamagnetic iron oxide (SPIO) nanoparticles or perfluorocarbon (PFC) emulsion to render monocytes detectable [74] (Fig. 3). Iron oxide nanoparticles have been developed to label and image monocytes in patents with cutaneous inflammation without affecting their viability or function, and show reliable safety for its application in humans [75]. By contrast, the ^19^F probe functions as a tracer agent in that ^19^F MRI directly detects the ^19^F nuclei that are associated with the labelled cells, unlike iron oxide nanoparticles, which are detected through their indirect effects on the surrounding water protons [74]. Researchers have successfully reflected macrophage infiltration with ^19^F MRI in pig myocardial infarction models, highlighting the potential of ^19^F MRI to monitor the inflammatory response after acute myocardial infarction (AMI) [76]. Other imaging techniques, like positron emission tomography (PET) or single photon emission computed tomography (SPECT), can also be applied to evaluate monocyte trafficking in disease models like rheumatoid arthritis [77] and atherosclerosis [78] in vivo.

### The interaction between NPs and neutrophils

Neutrophils are another group of cellular components of the innate immune system, possessing limited capacity for biosynthetic activity and with a significant role in resistance against extracellular pathogens in acute inflammation [81]. Neutrophiles account for 50–70% of all circulating leukocytes in humans and are characterized by a segmented nucleus with their cytoplasm enriched with multiple granules and secretory vesicles [82]. Originally, neutrophils occur from the hematopoietic cords located in venous sinuses in the bone marrow, and are derived from the default myeloid progenitor cells. Before developing mature and moving into circulation, neutrophils need to pass through three population pools in the bone marrow: stem cell pool, mitotic pool, and postmitotic pool [83] under the regulation of a group of transcription factors (GATA-1, Egr1, PU1, HoxB7, STAT3, C/EBPα-ζ), proteins (neutrophil elastase, S100A8, S100A9), and receptors (granulocyte–macrophage colony stimulating factor receptor and *N*-formyl-methionyl-leucyl-phenylalanine receptor) [82, 84], and finally, these mature cells are available for release and form reservoirs of mature neutrophils [85]. During inflammation, neutrophils in the circulation can be recruited by different adhesion molecules like P- and E-selectins and then, after the commonly recognized steps of recruitment (tethering, rolling, adhesion, crawling and transmigration), accumulate at the inflammatory region [86]. Upon activation and recruitment, these cellular components can eliminate pathogens by multiple means including phagocytosis, degranulation and releasing neutrophil extracellular traps etc., [87]. It is currently well-recognized that neutrophils are the front-line troopers of the innate immune system that can scavenge invaders and modulate innate inflammation. Besides, evidence shows that they also contribute to the onset and development of the adaptive immune reaction, endowing them a more complex role in the immune system [82]. With their significant roles in the pathogenesis of various inflammatory diseases, researchers have designed different types of nanoparticles to target neutrophils in order to modulate the inflammatory microenvironment and alleviate inflammation in disease models (Table 2). Mechanisms of their effects include modulating neutrophil migration, depleting inflammation-related neutrophils, neutrophil-based drug delivery, neutrophil biomimetic techniques etc.

**Table 2:** Interaction between nanoparticles and neutrophils

NP carriers	Functional molecules	Function	Diseases	References
Poly(lactide-coglycolide) (PLG) NPs	–	Modulating neutrophil migration	Spinal cord injury	[80]
Polystyrene (PS) NPs	Fluorescent carboxylate	Modulating neutrophil migration	Acute lung injury	[88]
PLG NPs	–	Modulating neutrophil migration	Autoimmune encephalomyelitis	[89]
Albumin NPs	DOX	Depleting activated neutrophils	Acute lung inflammation and ischemic stroke	[90]
Polydopamine-poly (ethylene glycol) NPs	DNase-1	Suppressing neutrophil proliferation and activity	Sepsis	[93]
Albumin NPs	Piceatannol	Neutrophil-based drug delivery	Acute lung injury	[94]
Albumin NPs	TPCA-1	Neutrophil-based drug delivery	Acute lung inflammation; bacterial infection	[96]
DGL NPs modified with PGP	Catalase	Neutrophil-based drug delivery	Cerebral ischemia	[97]
PLGA NPs	Neutrophil membrane coating	Neutralizing proinflammatory cytokines	Rheumatoid arthritis	[98]
Coenzyme Q NPs	Neutrophil membrane coating	Suppressing oxidative damages	Ischemia–reperfusion injury	[99]
Neutrophil membrane-derived nanovesicles	TPCA-1	Nanovesicle-based drug delivery	Acute lung inflammation	[100]
Neutrophil membrane-derived nanovesicles	Piceatannol	Nanovesicle-based drug delivery	Sepsis	[101]

#### Modulating neutrophil migration

Neutrophils can adhere to vascular endothelium and migrate into lesional tissue during inflammation process, which plays a primary role in a variety of inflammatory diseases [82]. Thus, it is a promising strategy to hamper the migration cascade to alleviate inflammation. Researchers have discovered that certain drug-free nanoparticle formulas have the ability to inhibit the migration of inflammation-related neutrophils. Park et al. revealed that intravenously (i.v.) administrated poly(lactide-coglycolide) (PLG) nanoparticles could be internalized by neutrophils in the circulation, which led to the reprogramming of neutrophil-related immune response [80]. In mouse models of traumatic primary spinal cord injury (SCI), after treated with PLG nanoparticles, the overall accumulation of neutrophils at the injury reduced by fourfold and fibrotic and gliotic scarring was significantly reduced by threefold comparing with sham group, indicating a proregenerative microenvironment that supports regeneration and functional recovery was formed [80]. Besides, they also found that the number of neutrophils accumulated in the spleen was significantly increased, showing an alteration in the migration process of innate immune cells. Similar strategy has also been applied in models of acute lung injury [88] and experimental autoimmune encephalomyelitis [89]. With more neutrophils migrating to the spleen rather than inflammatory leision, disease severity was significantly decreased. Taken together, these results showed that synthetic nanoparticles could modulate the migrating patterns of neutrophils under inflammation backgrounds and may be utilized to ameliorate inflammatory diseases in the future.

#### Depleting inflammation-related neutrophils

**Fig. 4:**
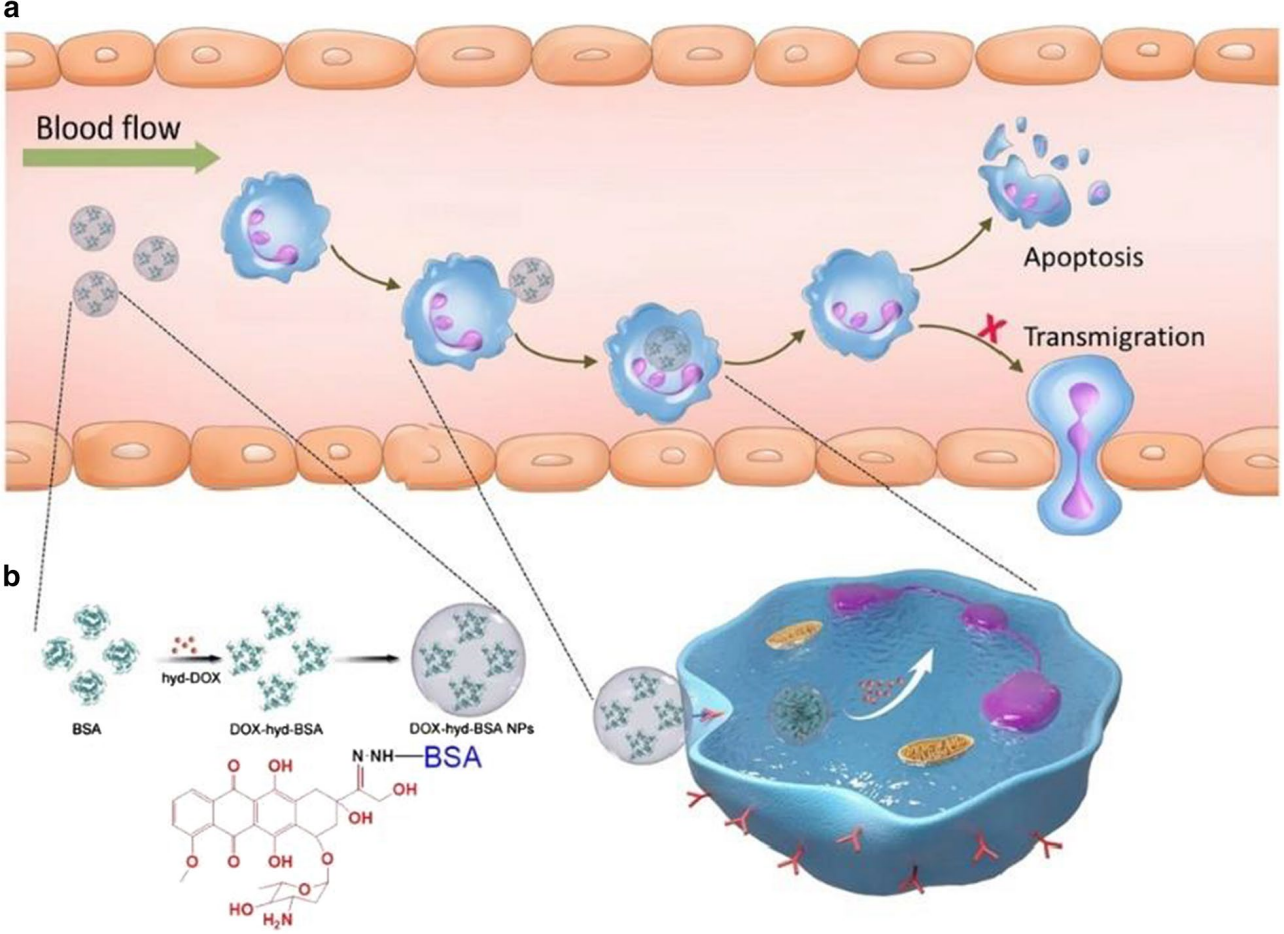
DOX conjugated bovine serum albumin (BSA) NPs can target proinflammatory neutrophils and induce their apoptosis in situ. DOX is conjugated to NPs through a pH-labile hydrazone bond, which can be cleaved by the low PH environment in activated neutrophils to release DOX and induce neutrophil apoptosis. Reprinted with permission [90]. Distributed under a Creative Commons Attribution NonCommercial License 4.0

Neutrophil accumulation is a major part of acute immune reaction, and the large amount of activated neutrophil population in the inflammatory region plays a pivotal role in the onset of inflammatory diseases. Different strategies have been developed to alleviate the severity of local inflammation either through depleting local neutrophils or suppressing the proliferation and activity of these cells. One type of pH-responsive albumin nanoparticles conjugated with doxorubicin (DOX) have been reported to have the ability of specifically targeting and depleting activated neutrophils [90]. DOX was conjugated to protein nanoparticles via a pH-labile hydrazone bond. After i.v. administration of these nanoparticles, they were internalized by neutrophils responded to infections or tissue injury, and the hydrazone bond would be cleaved by the acid environment in activated neutrophils to release DOX, which then induced the apoptosis of neutrophils. Such nano-formula showed therapeutic effects in mouse models of acute lung inflammation and ischemic stroke (Fig. 4). A more recent research focused on the neutrophil-related inflammation and cytokine storms caused by coronavirus. As disease severity progresses in patients with coronavirus disease 2019 (COVID-19), elevated amounts of cell-free DNA (cfDNA) and pro-inflammatory cytokines are released, which then mediated multiple organ failure [91, 92]. Researchers found that exogenously administered DNase-1-coated polydopamine-poly (ethylene glycol) nanoparticles could suppress the coronavirus-mediated neutrophil activities and the following cytokine storm [93]. With DNase-1 formulations treatment, mouse models of sepsis showed a significant reduction in number of neutrophil cell counts and levels of different inflammatory cytokines, accompanied by an increase in overall survival comparing with untreated groups.

#### Neutrophil-related drug delivery techniques

Similar to the monocyte/macrophage related hitchhiking strategies, neutrophils can also be used as transport carriers to deliver therapeutic agents. Wang et al. observed that denatured albumin protein nanoparticles (albumin NPs) could be internalized by neutrophils and could be used to deliver therapeutic drugs [94]. In vivo experiments showed that after such nanoparticles coated with dyes were up taken by neutrophils, unconjugated dyes would be observed to diffuse in these cells, indicating that these molecules were released from NPs upon internalization [95]. Besides, they also discovered that albumin NPs could only be up taken by neutrophils that were activated and adhered to endothelium, then these neutrophils would take them across the blood vessel barrier and accumulate in inflamed tissue [96]. Further study confirmed that such specifity was associated with the Fcγ RIII receptors on the surface of neutrophils. These results showed that drug delivery targeting neutrophils could be applied in the treatment of inflammatory diseases, and the therapeutic effects of this strategy have been proved in disease models of acute lung inflammation and bacterial infection [94, 96]. Researchers loaded albumin NPs with TPCA-1 (2-[(aminocarbonyl)-amino]-5-(4-fluorophenyl)-3-thiophenecarboxamide), an NF-κB inhibitor to treat a mouse model of acute lung inflammation. Inflammatory parameters including neutrophil infiltration and cytokine release were significantly decreased in the treatment group, and NPs also improved lung integrity to prevent lung edema [96]. The same nanoparticle formula loaded with cefoperazone acid (Cefo-A) was tested in *P. aeruginosa* infected models and the NPs dramatically declined bacterial proliferation by three folds compared with free drug administration [96]. Apart from these, Zhang et al. utilized cross-linked dendrigraft poly-l-lysine (DGL) nanoparticles to deliver catalase in a mouse model of cerebral ischemia. The nanoparticles were modified with the targeting ligand Pro-Gly-Pro (PGP), which provided them with high affinity to neutrophils. The infarction volume in treatment group was obviously decreased and the underlying mechanism was found related to the inhibition of ROS-mediated apoptosis [97]. Taken together, these results provide a new strategy to deliver therapeutics across the blood barrier to inflammatory lesions through neutrophil hijacking.

#### Neutrophil membrane-coated nanoparticles

**Fig. 5:**
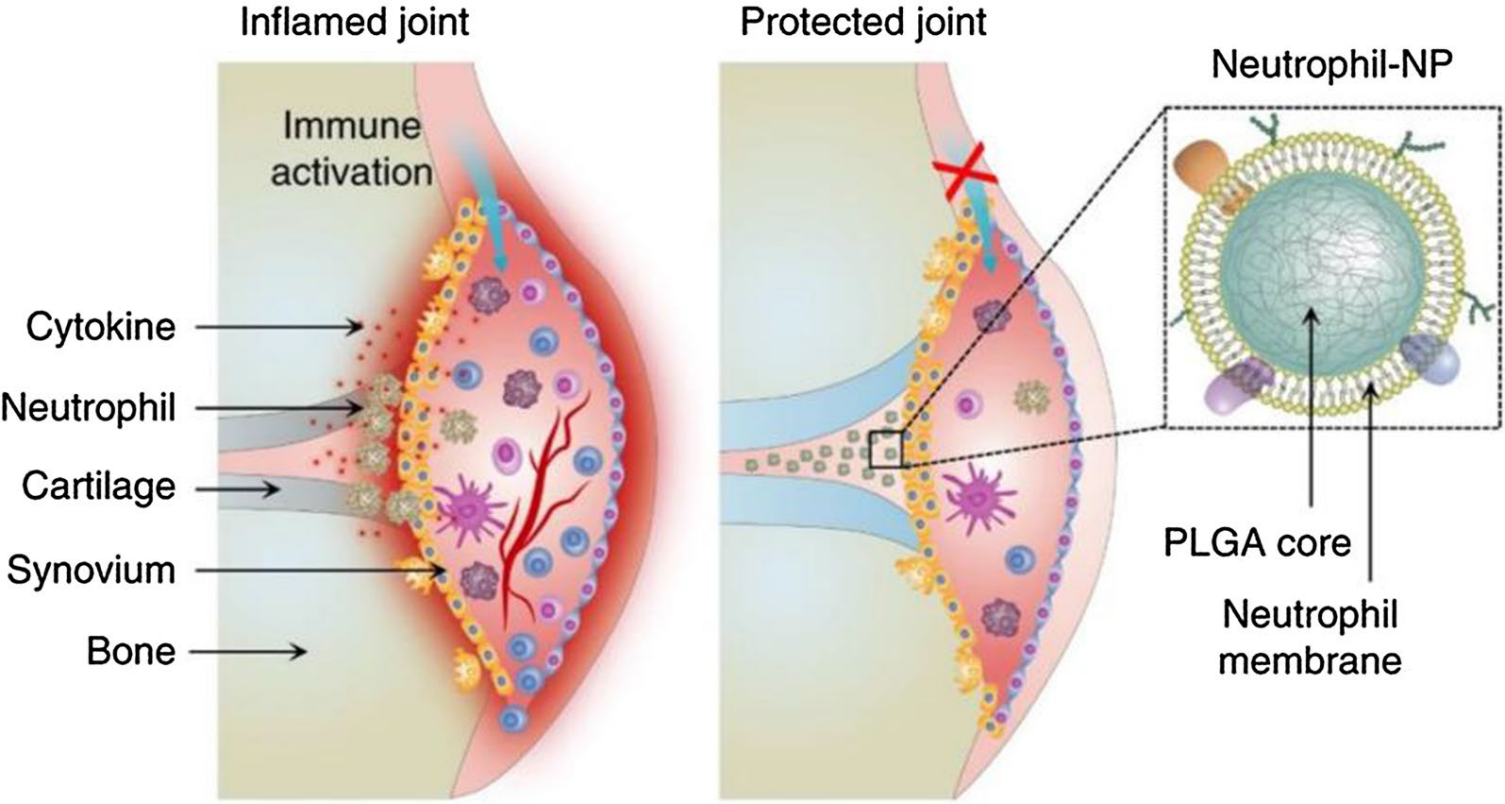
Schematic illustration of neutrophil-membrane coated polymeric NPs for the treatment of rheumatoid arthritis. Such biomimetic NPs inherit the natural characters of source cells, which can bind with inflammatory molecules, suppress synovial inflammation and provide strong chondroprotection effects. Reprinted with permission [98]. Copyright 2018 Springer Nature

Neutrophil membrane-coated nanoparticles inherit multiple characters of the natural neutrophils. They can escape from immune surveillance, neutralize pathological molecules and target inflamed tissue etc. Therefore, neutrophil mimicking nanoparticles have been regarded as promising therapeutic platforms for the treatment of inflammatory diseases [68, 98]. Zhang et al. designed neutrophil membrane-coated nanoparticles by fusing neutrophil membrane onto polymeric cores to treat rheumatoid arthritis [98]. Given the exterior antigenic and membrane associated functions inherited from the source cells, these nanoparticles could neutralize proinflammatory cytokines, suppress synovial inflammation, target deep into the cartilage matrix, and provide strong chondroprotection against joint damage in a mouse model of collagen-induced arthritis and a human transgenic mouse model of arthritis [98] (Fig. 5). Liu et al. developed a neutrophil membrane-enveloped Coenzyme Q (N-NP_CoQ10_) nanoparticle strategy for Ischemia–reperfusion (I/R) injury treatment. Results showed that N-NP_CoQ10_ nanoparticles administration exhibited synergistic protective effect against I/R injury, which significantly reduced oxidative damage in vitro and in vivo, inhibited renal cell apoptosis, attenuated inflammatory response in renal I/R injury model, and finally improved renal function of I/R injury mice [99]. Such formula provides a novel way of delivering anti-oxidant agents that can suppress oxidative damages. Apart from that, nanovesicles derived from neutrophil membrane can also be utilized in targeted drug delivery. Researchers collected nanovesicles from HL-60 cells which are neutrophil-like cells that arise after differentiation induced by chemicals [95] and loaded them with TPCA-1. Then these nanovesicles were administrated in an LPS-induced acute lung inflammation model. Results showed that neutrophil infiltration and the amount of TNF-α and IL-6 significantly decreased in the treatment group compared with free drugs and nanovesicles derived from red blood cells [100], suggesting that cell membrane-derived nanovesicles can be novel drug carriers targeting inflamed tissue. Similar strategy also showed therapeutic effects in an LPS-induced mouse sepsis model. Piceatannol-loaded nanovesicles were injected into LPS-induced mouse sepsis model and systemic inflammation in the lung, liver and kidney was ameliorated because of the reduction of neutrophil infiltration [101].

### The interaction between NPs and lymphocytes

Immune cells are derived from the hematopoietic stem cells (HSCs) in the bone marrow. HSCs primarily differentiate into common myeloid progenitor (CMP) and common lymphoid progenitor (CLP) cells. While CMP cells are the origins of granulocytes, macrophages, megakaryocytes, erythrocytes etc., CLPs give rise to various lymphocytes, with T and B lymphocytes as the main components and major effectors of the adaptive immunity [102]. T cell maturation is happened in the thymus after multipotent progenitors leave the bone marrow and move to the thymus, which includes complex processes like T cell receptor gene rearrangement and surface molecule (CD3, CD4, CD8 etc.,) expression. Mature T lymphocytes include effector T (T_E_) cells, regulatory T (T_reg_) cells and memory T (T_M_) cells, and can be classified into CD4^+^ T cells and CD8^+^ T cells according to their surface markers [103]. The CD4^+^ T lymphocytes, also called helper T (Th) cells, can modulate different immune reactions through the secretion of cytokines, while the CD8^+^ T cells are also named cytotoxic T lymphocytes (CTL) which are the main effectors of cellular immunity process. Unlike effector T lymphocytes, Treg cells act as negative modulator of the immune system which can inhibit the activity of effector cells [104] and T_M_ cells are the primary executors of immune memory, which is also an important part of the immune response [105]. B cell maturation is directly started in the bone marrow. After activation by antigens in the peripheral lymphoid tissue with or without the help of T cells, B lymphocytes can differentiate into plasma cells which can secret multiple antibodies and induce the process of humoral immunity [106], as well as memory B cells that respond quickly when meeting with the same antigen again [107]. Lymphocytes play a key role in the adaptive immune reaction, and a variety of nanoparticle platforms have been developed to modulate the immune system targeting these cellular components for the management of inflammatory diseases. Mechanisms include inducing T cell-related immune tolerance, depleting auto-reactive T lymphocytes and modulating B cell-related immune responses (Table 3).

**Table 3:** Interaction between nanoparticles and lymphocytes

NP carriers	Functional molecules	Function	Disease models	References
Poly(lactide-co-glycolide) (PLG) NPs	Ovalbumin antigen	Inducing Th2-related immune tolerance	Allergic airway inflammation	[108]
PLG NPs	Myelin antigen	Inducing Th/Th17-related immune tolerance	experimental autoimmune encephalomyelitis (EAE)	[109]
Pegylated iron oxide NPs	Autoimmune disease-relevant peptides-major histocompatibility complex class II (pMHCII) molecules	Inducing Treg cell expansion	Non-obese diabetes (NOD); EAE; collagen-induced arthritis (CIA)	[111]
Gold NPs	Autoimmune disease-relevant peptide-major histocompatibility complex (pMHC) molecules	Inducing Treg cell expansion	NOD	[112]
Gold NPs	(1′H-indole-3′-carbonyl)-thiazole-4-carboxylic acid methyl ester (ITE), β cell antigen proinsulin	Inducing tolerogenic phenotype in dendritic cells; inducing differentiation and expansion of FoxP3^+^ Treg cells	NOD	[110]
PLGA NPs	Mannan/ApoB peptide	Inducing Foxp3^+^ Treg cell expansion	Allergic airway inflammation	[114]
Curcumin NPs	–	Inducing Treg cell expansion; modulating gut microbiota	Dextran sulfate sodium-induced colitis	[116]
Gold NPs	Methotrexate (MTX)	Depleting auto-reactive T cells	Psoriasis	[121]
PEGylated PLGA NPs	Eggmanone	Inhibiting CD4^+^ T cells	Rheumatic diseases	[123]
Superparamagnetic iron oxide (SPIO) NPs	Tumorigenicity 2 (ST2) antibody	Inhibiting CD4^+^ T cells	OVA-induced lung inflammation	[125]
Siglec-engaging Tolerance-inducing Antigenic Liposomes (STALs)	CD22, synthetic citrullinated antigen	Inhibiting auto-reactive B cells	Rheumatoid arthritis	[128]
Poly (DL-lactic-co-glycolic acid) NPs	Synthetic citrullinated peptide, complement-activating lytic peptide	Depleting auto-reactive B cells	Rheumatoid arthritis	[129]
Iron-oxide NPs	Anti-CD20 monoclonal antibody	Depleting auto-reactive B cells	Multiple sclerosis	[130]

#### Inducing T cell-related immune tolerance

**Fig. 6:**
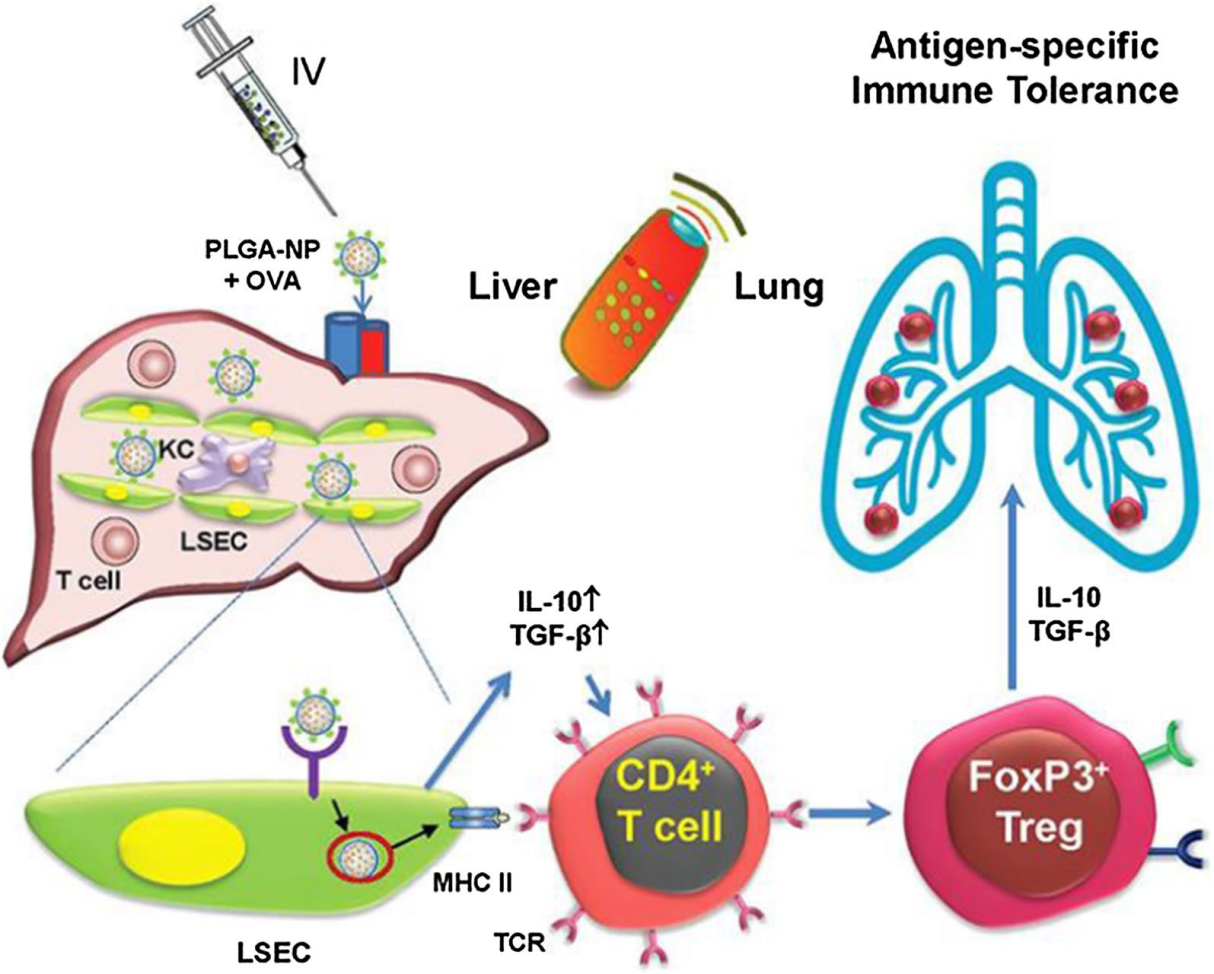
Immune tolerance can be induced by delivering ovalbumin (OVA) to the liver through PLGA NPs. NPs are decorated with ligands that targets sinusoidal endothelial cells (LSECs) and induce the differentiation of Foxp3^+^ Treg cells, which can alleviate airway inflammation in OVA sensitized animals. Reprinted with permission [114]. Copyright 2019 American Chemical Society

Auto-reactive immune response is a major concern in the pathogenesis of various inflammatory diseases. Therefore, the reestablishment of immune tolerance is a primary goal for the treatment of such conditions. Relevant antigen delivery has been considered as a reliable method to induce immune tolerance. Researchers have utilized poly(lactide-co-glycolide) NPs containing encapsulated antigen [PLG(Ag)] to treat Th2-mediated allergic airway inflammation. PLG(Ag) NPs were well tolerated and effectively inhibited ovalbumin (OVA)-induced Th2 responses and airway inflammation both prophylactically and therapeutically [108]. Similar strategy has also been introduced in the management of Th1/Th17-mediated autoimmune disease [109]. Besides, several regulatory T (Treg) cell subsets like the FoxP3^+^ Treg cells can mediate immune tolerance through their interactions with different cellular components in the immune system, so researchers have tried to develop nanoparticle platforms focused on Treg cell expansion in autoimmune disease models [110]. Clemente-Casares et al. showed that that systemic delivery of nanoparticles coated with autoimmune disease-relevant peptides which were linked to major histocompatibility complex class II (pMHCII) molecules would trigger the differentiation and expansion of antigen-specific regulatory CD4^+^ T cell type 1 (T_R_1)-like cells in different disease models like non-obese diabetes (NOD), experimental autoimmune encephalomyelitis (EAE) and collagen-induced arthritis (CIA) [111]. Such administration leaded to restoration of established autoimmune phenomena and further study proved that the cell surface phenotype, transcriptional profile, cytokine secretion pattern and function of these T_R_1-like cells were consistent with murine T_R_1-like CD4^+^ T cells and remarkably similar to human T_R_1 cells. They also elucidated key roles for prior autoantigenic experience and IFNγ- and IL-10-expression level in the development of autoreactive T_R_1 cells and shed light on a cascade of cellular interactions related with the therapeutic effects of pMHC NPs including T-bet, IFNγ, c-Maf /IL-10 and IL-21 [111]. In a more recent study, they tried to figure out the physical/chemical parameters impacting pharmacodynamics of such nanoparticle formulas and found that pMHC density was responsible for the Treg-triggering potency of these compounds which meant that total pMHC dose conjugated to nanoparticles was associated with their Treg-expanding properties [112]. They also introduced a novel and simple manufacturing process that could consistently yield highly stable and potent metal oxide-based Treg-triggering/expanding nanomedicines [112]. Another group constructed gold nanoparticles containing the aryl hydrocarbon receptor (AhR) ligand 2-(1′H-indole-3′-carbonyl)-thiazole-4-carboxylic acid methyl ester (ITE), and the β cell antigen proinsulin (NP_ITE+Ins_) to treat NOD mice [110] and the spontaneous development of Type 1 diabetes (T1D) was suppressed. They uncovered that NP_ITE+Ins_ functioned through inducing a tolerogenic phenotype in dendritic cells (DCs) and increasing the differentiation of FoxP3^+^ Treg cells in an Socs2/NF-κB dependent manner [110]. Apart from these formulas, there are also strategies that directly introduce antigens into natural tolerogenic environments, like the liver [113], where antigen presentation promotes tolerance to self or foreign antigens [114]. Liu et al. used poly (lactic-co-glycolic acid) (PLGA) nanocarriers to deliver the murine allergen, ovalbumin (OVA), to the liver [114]. The NPs were decorated with ligands that targets the specialized tolerogenic liver sinusoidal endothelial cells (LSECs) which are capable of generating regulatory T-cells (Tregs). In vivo experiments showed that OVA-NPs were almost exclusively delivered to the liver after i.v. administration and could significantly alleviate airway inflammation in OVA sensitized and challenged animals. OVA-NPs induced the differentiation of Foxp3^+^ Treg cells, together with abundant TGF-β, IL-4 and IL-10 production and suppression of anti-OVA IgE responses (Fig. 6). Naturally-sourced compounds like curcumin have drawn the interest of pharmacological development. Curcumin is a kind of hydrophobic polyphenol extracted from turmeric, a traditional Indian spice [115]. It has recently been expected for the treatment of inflammatory diseases and cancers given its multi-targeted activities and anti-inflammatory function [115–117]. However, clinical application of curcumin has been limited due to low oral bioavailability, so novel administration method needs to be developed to improve the absorption rate. Ohno and his team assessed the therapeutic effects of nanoparticle curcumin on the development of inflammatory bowel diseases (IBD) and showed that nanoparticle curcumin could attenuate body weight loss, disease activity index, histological colitis score and improve mucosal permeability in dextran sulfate sodium (DSS)-induced colitis model, accompanied by increased expansion of CD4^+^ Foxp3^+^ regulatory T cells [116].

#### Depleting auto-reactive T lymphocytes

**Fig. 7:**
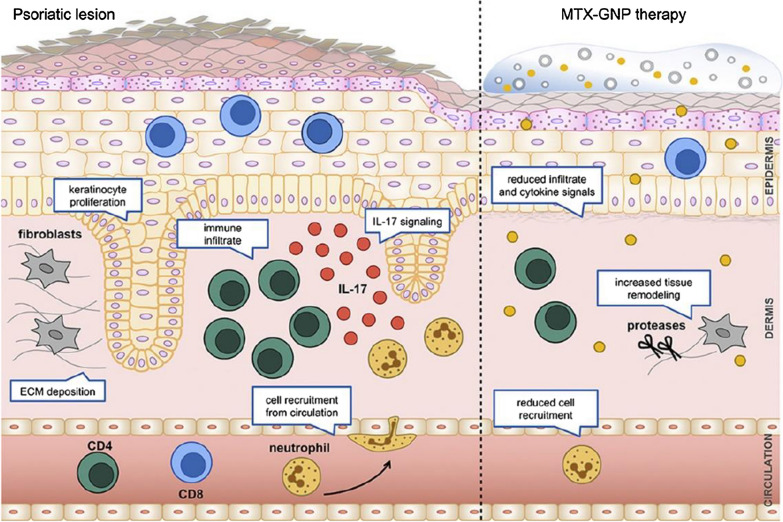
Gold nanoparticles (GNPs) coupled with MTX show significant antiinflammatory effects in psoriasis. γδ T and CD4^+^ T cells are significantly reduced in skin lesions and skin hyperplasia was inhibited, accompanied by superior tissue remodeling. Reprinted with permission [121]. Copyright 2020 Elsevier

Autoreactive T lymphocytes play important roles in the onset of a variety of inflammatory diseases like rheumatism and acute inflammation. Several nanoparticle platforms have been tried for the direct depletion or inhibition of these pathogenic lymphocytes. Strategies include cytotoxic drugs delivery, cell signaling inhibition and cell receptor suppression etc. Methotrexate (MTX) is an antiproliferative drug widely used for inflammatory diseases and cancers [118–120]. It can inhibit cell proliferation and impair the activation and migration of leukocytes, such as T cells, macrophages, and neutrophils [120]. Özcan et al. showed that gold nanoparticles combined with MTX demonstrated superior antiinflammatory efficacy than MTX alone in an imiquimod-induced psoriasis mouse model [121]. The γδ T and CD4^+^ T cell count was significantly reduced and skin hyperplasia was inhibited compared with calcipotriol-betamethasone treatment group, accompanied by profound tissue remodeling [121] (Fig. 7). Hedgehog (Hh) signaling plays a key role in the activation of T cells and has been implicated as a therapeutic target of inflammatory diseases in recent years [122]. Researchers have leveraged polymeric NPs to targeted deliver eggmanone (Egm), a specific Hh inhibitor, to CD4^+^ T cells via the decoration of anti-CD4 F(ab′) antibody fragments. The novel delivery system targeted CD4^+^ T cells with high specificity and showed a significant inhibitory effect on CD4^+^ T cell responses, presented by a reduction of cytokine secretion and helper T cell activation [123]. Group 2 innate lymphoid cells (ILC2s) are a group of type 2 (T2) inflammatory cells which are implicated in the pathogenesis of asthma [124]. Wu and his teammates have investigated the effects of targeting suppression of tumorigenicity 2 (ST2), the ILC2 receptor, using nanotechnology [125]. They treated OVA-induced mice with superparamagnetic iron oxide (SPIO) NPs conjugated with anti-ST2 antibody. Lung inflammation induced by OVA was significantly mitigated and further study showed that anti-ST2-conjugated NPs reduced the ability of ILC2 to produce IL-5 and IL-13, thereby inhibiting the differentiation and expansion of CD4^+^ T cells [125]. These studies also demonstrated that nanoparticle delivery platforms could improve the efficiency of monoclonal antibody, which may be clinical transformed as strategic tools in the future for drug delivery.

#### B lymphocyte-related immune modulation

B lymphocytes are executors of the adaptive immune system, which function through differentiation into plasma cells and secret antibodies after B cell receptors (BCR) binding to their matched antigens [126]. Auto-reactive B cells are implicated in the pathogenesis of several inflammatory diseases with the ability to secret autoantibodies. Presently, NP strategies related with B lymphocyte reaction in managing inflammatory conditions mainly focus on the direct targeting and inhibition/depletion of autoreactive B cells. For example, anti-citrullinated protein autoantibodies (ACPA) from memory citrulline-specific B cells are pathogenic factors of rheumatoid arthritis (RA) and among the hallmarks of RA [127]. Bednar et al. developed Siglec-engaging Tolerance-inducing Antigenic Liposomes (STALs) co-displaying CD22, an immune inhibitor, and synthetic citrullinated antigen (CCP STALs) to treat RA [128]. Results showed that CCP STALs could inhibit ACPA production from RA patients’ memory B cells in vitro, and were also effective in inducing tolerance to citrullinated antigens in mouse model [128]. Another team showed that poly (dl-lactic-co-glycolic acid) NPs decorated with synthetic citrullinated peptide and complement-activating lytic peptide could significantly reduce the ACPA production of B cells from peptide seropositive RA patients, indicating a potential to specifically target and deplete autoreactive B-cells for RA treatment [129]. B cell inhibition/depletion method can also be used for central nervous system (CNS) autoimmune diseases. Carnasciali et al. designed a nanotechnology-based cell-mediated drug delivery system which loaded myelin antigen-specific T cells with anti-CD20 monoclonal antibody binding NPs. Such formula could significantly deplete B cells in an animal model of multiple sclerosis (MS), both in the spleen and in the brain, and effectively ameliorated the disease course and pathology [130].

### The effects of NPs on inflammation-related molecules

Apart from cellular components of the immune system, various types of molecules also exert vital functions in the process of inflammation and have become the therapeutic targets of inflammatory diseases, including cytokines, receptors, enzymes, reactive oxygen species (ROS) and so on. These molecules also constitute a major part of the immune system. Immune cells interact with each other and modulate immune responses through the effectors they secret and the specific receptors that accept certain signals. Thus, adjusting the level of different inflammation-related molecules can be a reliable strategy to modulate the immune system, especially in the treatment of inflammatory diseases. As we have mentioned above, NPs can influence the level of cytokines by targeting their source cells and modulating their producing patterns [121, 125]. Besides, NPs can also interfere with inflammation-related molecules through direct interaction with them, like neutralizing effect, absorption, anergy etc., (Table 4).

**Table 4:** NP strategies targeting inflammation-related molecules

NP carriers	Functional molecules	Effects on inflammatory molecules	Disease models	References
Poly (lactic-co-glycolic acid) (PLGA) NPs	Neutrophil cell membrane coating	Neutralizing and absorbing pathogenic cytokines	Rheumatoid arthritis	[98]
PLGA NPs	Macrophage cell membrane coating	Neutralizing endotoxins and proinflammatory cytokines	Sepsis	[79]
Dopamine NPs	Melanin	Scavenging reactive oxygen species (ROS) and reactive nitrogen species (RNS)	Osteoarthritis	[131]
Polyethylene glycol (PEG) conjugated dopamine NPs	Melanin	Scavenging ROS and RNS	Ischemic stroke	[133]
PEG NPs	Bilirubin	Scavenging ROS	Colon inflammation	[135]
PEG NPs	Bilirubin	Scavenging ROS	Pancreatic islet xenotransplantation	[136]
Polystyrene NPs	Surface decoration: sulfon (OSO_3_); carboxyl (COOH); amine (NH_2_)	Inhibiting the activity of myeloperoxidase	–	[138]
Polydopamine-poly (ethylene glycol) NPs	DNase-1	Eliminating cell-free DNA (cfDNA)	Severe acute respiratory syndrome coronavirus 2 (SARS-CoV-2)-mediated cytokine storms	[93]
PLGA NPs	Metformin hydrochloride	Downregulating the level of IL-1β and TNF-α	Periodontitis in diabetic background	[140]
Silk fibroin NPs	–	Downregulated the level of pro-inflammatory cytokines	Inflammatory bowel disease	[141]

**Fig. 8:**
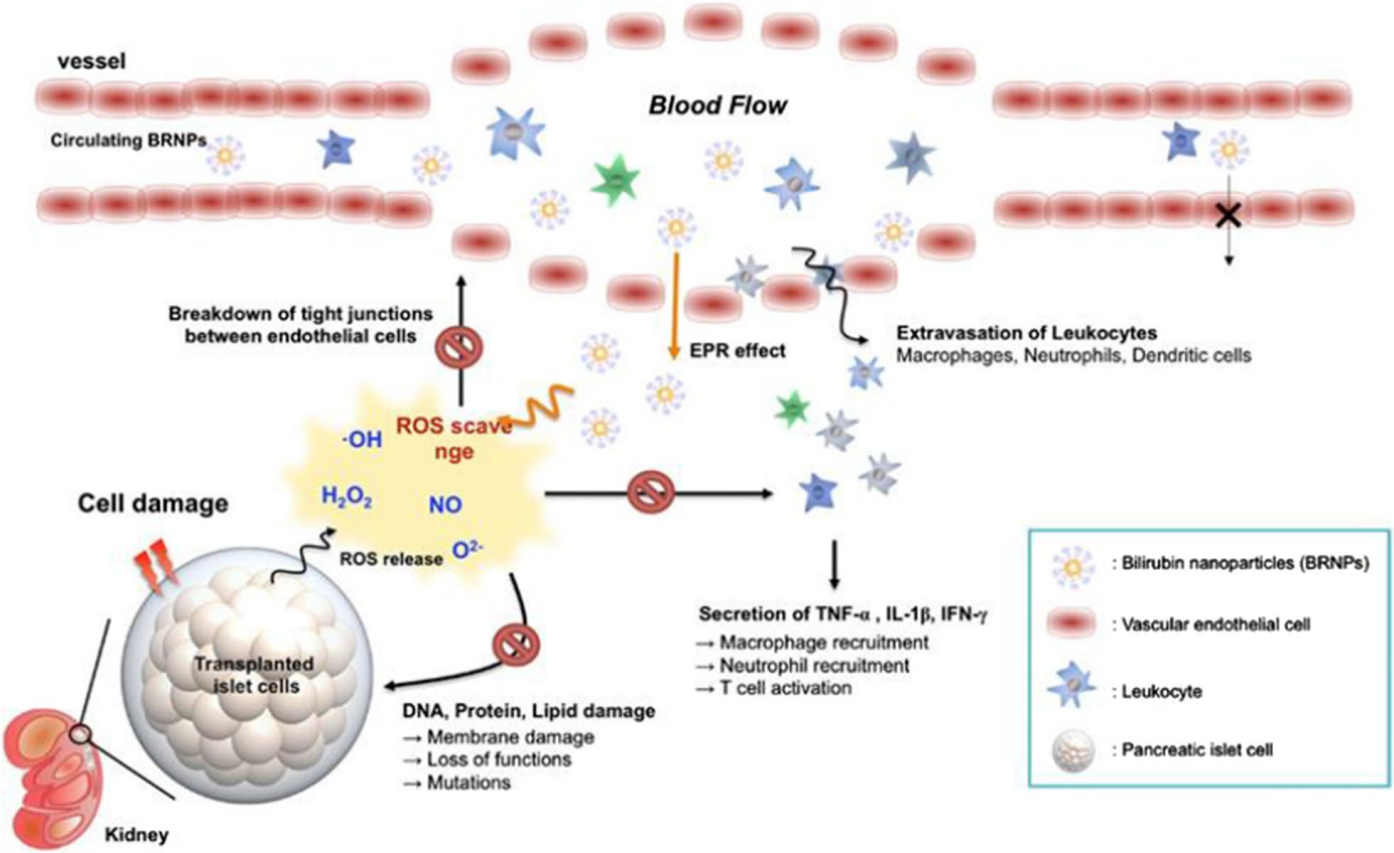
PEGylated bilirubin nanoparticles (BRNPs) can protect transplanted islet cells from inflammation-related oxidative stress. Excessive ROS molecules could be produced by transplanted islets upon cell damage. These ROS molecules recruit inflammatory neutrophils and macrophages and produce more ROS and cytokines, which forms a positive feedback loop. BRNPs can inhibit this process through scavenging the ROS molecules and show satisfying therapeutic effects. Reprinted with permission [136]. Copyright 2017 Elsevier

In recent years, cell membrane-coated NPs have aroused researchers’ interest as novel therapeutic platforms [68]. Given the antigenic profile inherited from source cells, membrane-coated NPs are able to act as decoys that can neutralize and absorb heterogeneous and complex pathological molecules regardless of their structural specificity [98]. Zhang and his team discovered that neutrophil membrane-enveloped PLGA NPs could bind and neutralize IL-1β and TNF-α cytokines in a mouse model of collagen-induced arthritis and a human transgenic mouse model of arthritis [98], accompanied by remarkably amelioration of joint damage and suppression of overall disease severity. A similar macrophage membrane-coated NP has also been used as detoxicant to neutralize endotoxins and proinflammatory cytokines in a model of sepsis [79]. Reactive oxygen species (ROS) and reactive nitrogen species (RNS) have been implicated in the pathogenesis of inflammatory diseases, which can cause uncontrollable oxidative stress that induces DNA, lipid, and protein damage, as well as cytotoxicity [131]. Researchers have tried to develop nanotechnology-based platforms as emerging anti-oxidative agents for the management of reactive oxygen and nitrogen species (RONS) [132, 133]. Zhong et al. explored dopamine melanin (DM) NPs as a novel RONS scavenger in a rat model of osteoarthritis. Results showed that DM NPs had a strong ability to eliminate a wide range of RONS including superoxides, hydroxyl radicals, and peroxynitrite, and also inhibit the expression of pro-inflammatory cytokines like IL-1β, leading to ideal anti-inflammatory and chondro-protective therapeutic effects [131]. A similar strategy has also been applied by Liu et al. to treat ischemic stroke which exhibited unique multi-antioxidative, anti-inflammatory, and biocompatible features [133]. In addition to melanin NPs, bilirubin nanomedicines have recently been reported with a strong anti-oxidative ability and have been tested in a number of inflammation-related disease models (Fig. 8) [134–136]. Studies showed that bilirubin NPs are effective scavengers of ROS and could protect tissue cells from oxidative stress, accompanied by a reduction of inflammatory cytokines production [135, 136]. Myeloperoxidase (MPO) is one of the lytic enzymes released by activated neutrophils and has been demonstrated to be a local mediator of tissue damage and the resulting inflammation in various inflammatory diseases [137]. Sanfins et al. noticed that polystyrene NPs could interfere the activity of neutrophil-released MPO, and such effect could be affected by different chemical decoration on the surface of NPs and by the presence of other proteins like bovine serum albumin (BSA) [138], providing a potential strategy to manage the activity of MPO in pathological process. During the process of severe acute respiratory syndrome coronavirus 2 (SARS-CoV-2)-related neutrophil activation and the cytokine storm, large amounts of cell-free DNA (cfDNA) will be produced and encapsuled in neutrophil extracellular traps (NETs), leading to severe organ failure in these patients [93, 139]. Based on such theory, researchers have constructed recombinant DNase-1-coated NPs targeting cfDNA for this condition and achieved significant suppression of the SARS-CoV-2-mediated cytokine storm [93]. Besides, there are also studies reporting that NPs can modulate the level of multiple cytokines in managing inflammatory conditions. For example, metformin hydrochloride-loaded PLGA NPs can suppress the level of IL-1β and TNF-α [140]. Silk fibroin-based NPs downregulated the level of pro-inflammatory cytokines (e.g., IL-1β, IL-6, IL-12 and IL-17) in a rat model of inflammatory bowel disease [141, 142]. However, the underlying mechanisms, though not fully elucidated, may be associated with NPs’ influence on immune cells rather than cytokines themselves, which need to be further verified. The complement system, composed of a variety of soluble and membrane-bound proteins, is another essential part of the immune system and plays a pivotal role in non-specific defence against pathogens, particulates, self-damaged and aged cells [143]. Activation of the complement system is a complex proteolysis-based cascade, which proceeds through mainly three pathways, namely alternative, classical and lectin pathways [144]. Currently, a large number of NP formulas have been designed to activate or interact with the complement system through all the three pathways, the mechanisms of which have been reviewed elsewhere [143]. But few of them has been linked with the management of inflammatory diseases, which needs to be further explored. Taken together, these phenomena implied that NPs can affect the immune system not only at cellular level, but also through direct interaction with released immune effectors.

### Other effects of NPs on the immune system

In addition to the above interactions between NPs and the immune system, there are other mechanisms through which NPs can influence immune responses, including interaction with dendritic cells, modulating nature killer (NK) cell activity and so forth.

Dendritic cells are an important group of antigen-presenting cells (APCs) linking innate and adaptive immune responses [145, 146]. Different NP platforms related to DCs have been developed given their powerful ability to present antigen and modulate immune reaction. DCs play a key role in the induction of immune tolerance, so it can be a promising strategy targeting DCs for the alleviation of inflammation. Yeste et al. engineered NPs to deliver β cell antigen proinsulin combined with a tolerogenic molecule, the aryl hydrocarbon receptor (AhR) ligand 2-(1′H-indole-3′-carbonyl)-thiazole-4-carboxylic acid methyl ester (ITE) in a mouse model of type 1 diabetes [110]. A tolerogenic phenotype in DCs was induced characterized by a dampened ability to activate inflammatory effector T cells and an increase in the differentiation of FoxP3^+^ Treg cells [110]. Another study showed that naturally-derived broccoli NPs could prevent activation of DCs and induce a tolerant phenotype in DCs in an adenosine monophosphate activated protein kinase (AMPK)-dependent manner [147]. Besides, researchers have developed cationic liposomes (CLs) to influence the efficiency of antigen-presenting of DCs. Results showed that CLs could elevate the lysosomal pH in DCs and reduce antigen degradation, leading to the promotion of cross-presentation and cross-priming of CD8^+^ T cell responses [148].

NK cells are classified as innate lymphoid cells and exert significant functions in the innate immune system. They are capable of eliminating abnormal cells through different ways like exocytosis of perforin, death-inducing enzymes and initiating of death receptor pathways [149]. Therefore, they can be utilized by NP strategies for the killing of undesired cells. For example, a novel NP formula, bispecific Au NPs (BiAb-AuNPs), which are generated through conjugating anti-HIVgp120 and anti-human CD16 IgG antibodies onto Au NPs, have been shown to significantly enhance the cytotoxic immune response of NK cells towards HIV-infected T cells [149]. Besides, another group developed a NP-based cell tracking method which used lanthanide-based down-conversion NPs (DCNP) coated with a ROS sensitive near infrared (NIR) dye, IR786s. Such nanomaterials were directly labeled on NK cells, and when these NK cell died, elevated ROS would trigger NIR-II signal, which linked fluorescent signal with cell viability of NK cells [150]. These nanotools show a strong potential for the application of NK cells related NP strategies in the treatment of inflammatory diseases.

Other mechanisms like adjusting neural-immune cells cross-talk, preventing hypoxia relevant apoptosis and modulating interaction between immune effector cells and non-immune cells have also been explored [60, 151], but their potential in treating inflammatory diseases still need to be further confirmed by in vivo studies.

## Perspectives and discussion

As we described above, nanoparticle platforms can interact with almost every component in the immune system and provide us with a novel tool for the treatment of immune-related diseases. Clinically, the management of inflammatory diseases largely relied on application of anti-inflammatory drugs like glucocorticoids or biologicals [152, 153], which aims at palliation and have considerable side effects given their systemic distribution and relatively weak specificity. In comparison, NPs can have a more accurate targeting effect, and with modulation of different properties of the NPs like size, shape, biodegradability, surface charge, and the combination of multiple functional molecules like antibody, cell membrane, enzyme etc., NPs can be granted with numerous therapeutic characters and infinite potentials for the amelioration of inflammation. In this review, we summarize the various interactions between NPs and components of the immune system and their possible application in the treatment of inflammatory diseases. These studies suggest that, with a known pathogenic mechanism of a certain disease, we can design NP formulars targeting the involved cellular or molecular components in the immune system and realize the accurate interference to the inflammatory environment.

However, application of NPs in immune modulation still faces with several challenges. Firstly, in some cases, modulatory NPs function through depleting or inhibiting immune cells to suppress overactive inflammatory reactions. Such strategy, on the other hand, may lead to immune dysfunction or immunodeficiency in the body, which increase the possibility of opportunistic infections and malignancy. Secondly, although we have developed NPs to interact with certain cells or molecules in the immune system, they may also affect healthy immune or non-immune cells in the organism, causing unwanted side effects that may not be discovered in pre-clinical data. Thirdly, there exists a nonnegligible gap between animal models and human body, so however ‘exhilarating’ the pre-clinical results maybe, NPs still need to be tested in clinical trials, and some of them may fail to exert immunomodulatory effects in human bodies [154]. Fourthly, NPs have been implicated as novel drug carriers in multiple studies. They can increase the stability and circulating time of the therapeutic agents, protect them from the quick clearance of the mononuclear phagocytic system and deliver drugs directly to the inflamed tissues. But such transporting strategy may also amplify the side effects of the agents, with longer circulating time and a more direct contact with the organism. These obstacles require further studies into the mechanisms behind the anti-inflammatory effects of NPs and systemic clinical trials to evaluate the biosecurity of such nanomaterials. We also need to know more about the physiological distinction between model animals and human bodies to better link the pre-clinical results to clinical effects. Moreover, researchers have proved that certain physical characteristics like size, shape, surface potential can influence the effects of NPs [5, 155], so adjusting the physical characters of NPs can be a promising policy to optimise the therapeutic effects and pharmacokinetics of the agents.

Given the rising incidence and heavy health burden of inflammatory diseases worldwide, it is necessary to develop advanced strategies for efficient disease intervention. Current trends towards the development of precision medicine have granted NP platforms with unlimited application potential. Nevertheless, although novel NP strategies have been emerging continuously, clinically approved nanotherapeutics are still pretty rare, with too many unknown factors involved in this frontier technology. NPs offer a variety of modifiable features like size, shape, charge, surface modification etc., which can be further customized for intelligent NP design. For example, NP charge is of importance in endosomal escape as well as muco-penetrating application, while targeting surface markers can be utilized for specific cell type uptake [156]. These features offer plenty of choices for the realization of multifunctional strategies. However, as the clinical demands and design considerations become more complicated, researchers tend to involve more manufacture elements into their formulas, which increase the unpredictability of their performance in clinical trials. Therefore, it is of great necessity to thoroughly investigate the NP design and the probable interactions with human body, which will be beneficial for the safety and specificity of these platforms, and also help to determine the most suitable NP strategies for a certain patient subgroup. Besides, industrial-scale production of NPs should meet the requirement of good manufacturing practice (GMP) to guarantee the consistency from batch to batch [157]. Despite all the challenges we meet, it is undeniable that NPs have switched the therapeutic target of inflammatory diseases from symptomatic relief to etiological treatment of inflammation with their ability to interact with almost every pathogenic components in the immune system as we described in this review. It is foreseeable that with proper design and rigorous safety evaluation, NP platforms will play a pivotal role in the bioimaging and therapy for the precision medicine of inflammatory diseases.

The translation of NPs from bench to bedside has always been a practical problem, especially in the treatment of inflammatory diseases. Although the actual FDA-approved NPs and those under clinical trials are still scarce, we can learn some common characters from these successful examples. Generally, most of the NP formulas that are approved for market or under clinical trials are polymeric NPs, liposomes or inert metal related delivery systems. For instance, Zilretta^®^, FDA approved agent for managing osteoarthritis, is a PLGA NP that can slowly release triamcinolone acetonide in the synovium [158]. CAL02 constructed by Combioxin SA company is a form of liposome containing sphingomyelin and cholesterol for toxin neutralization, and has completed the phase I clinical trial for pneumonia (NCT02583373) [159]. C19-A3 GNP, gold NP coupled with proinsulin peptide, is being tested for type 1 diabetes (NCT02837094) [160]. Such NP platforms share the common formula that a stable NP carrier with good biocompatibility is utilized for delivering certain therapeutic molecules. The safety, stability and well connection to therapeutic molecules value most for the carriers, which realize the increase of half-life or a controlled release of the functional components in the inflammatory regions. Such experience indicates that future NP platform designing should also follow the combination principle of safety, stability and effective treatment for maximizing the clinical translation potential.

In the past decade, several novel NP strategies have been introduced with unique characteristics and great application potential in biomedical field. They may implicate the future directions for NP-based immune modulation. For instance, cell membrane-coated NPs are emerging nanotools that can utilize the inherent ability of the sourced cells to influence the environment. Currently, cell membranes can be obtained from almost every kind of cells like erythrocytes, leukocytes, stem cells and even cancer cells [68]. The coating of different membrane grants NPs with a variety of functionalities, and together with all kinds of core compositions encapsuled, these NPs form a distinctive platform that has wide-ranging application possibility including drug delivery, imaging, photoactivatable therapy, detoxification etc. Metal–polyphenol network (MPN) is another example of the ‘combination strategy’ that comprises of polyphenols and multivalent metal ions. Metal provides polyvalent complexing, anti-bacteria and tissue repair promoting ability, while polyphenol functions as important antioxidants and anti-inflammation agents. MPN-based NPs have aroused the interest of researchers with their salient characters like structure flexibility, thermal stability and pH responsiveness, and have been developed into multiple NP platforms for the treatment of several diseases [161]. These examples suggest that future NP strategies will still focus on utilizing biochemical synthesis techniques to add different chemical or biological components to broaden their application range and improve existing structures. Nevertheless, such novel formulas also need to be treated with caution given their structure complexity and some important properties have not been studied in depth. Translation from laboratories to clinics still needs more comprehensive studies and clinical evaluation.

## Conclusion

The rapid development of nanotechnology has provided us with a new tool for the modulation of immune reaction, especially in the management of multiple inflammatory diseases. Currently, several immunomodulatory nanodrugs have been approved by FDA, and more are in the stages of pre-clinical study or clinic trials. With their unique structures, plasticity and stability, NPs show a great potential to target and accumulate in the inflamed tissues, and in comparison with the current anti-inflammatory agents which are mostly palliative, NPs are able to address the pathogenic factors of inflammatory diseases. However, our present understanding of NP interaction with immune system is still finite, and we can hardly predict the clinical performance of many NP formulas even with excellent pre-clinical results, so rigorous clinical trials and long-term safety evaluation are necessary. There is also need for the development of reliable techniques to monitor the therapeutic effects of NPs in the body. In general, immunomodulatory NPs have the potential to change the treatment patterns of inflammatory diseases with their salient characters, but there is still a long way to go before their actual translation into clinics.

## Data Availability

Not applicable.
